# External validation of the BAN‐ADHF diuretic‐resistance score in the DAPA‐RESIST clinical trial

**DOI:** 10.1002/bcp.70619

**Published:** 2026-05-20

**Authors:** Dominic M. Alfonso, Kieran F. Docherty, Matthew M. Y. Lee, Patrick B. Mark, Alex McConnachie, Joanna Osmanska, Bethany Stanley, Atefeh Talebi, Mark C. Petrie, John J. V. McMurray, Ross T. Campbell

**Affiliations:** ^1^ School of Cardiovascular and Metabolic Health University of Glasgow Glasgow UK; ^2^ Robertson Centre for Biostatistics, School of Health and Wellbeing University of Glasgow Glasgow UK

**Keywords:** dapagliflozin, diuretic resistance, heart failure, metolazone, SGLT2i, thiazide

## Abstract

Diuretic resistance in worsening heart failure (HF) is associated with poorer outcomes and, although common, can be challenging to identify. The recently developed BAN‐ADHF score can potentially identify patients with worsening HF at risk of diuretic resistance. We externally validated the BAN‐ADHF diuretic‐resistance score in a multicentre randomized trial comparing adjunctive dapagliflozin to metolazone in patients with HF and diuretic resistance. We calculated a seven‐variable modified BAN‐ADHF score at baseline and dichotomised patients by the median BAN‐ADHF score (8). We assessed loop diuretic efficiency (kg weight change per 40‐mg furosemide) and decongestion (weight, eight‐zone lung ultrasound B‐lines, clinical congestion score) over 96 h. A baseline score ≥8 identified lower loop diuretic efficiency (pooled difference −0.12 kg/40 mg, 95% CI −0.18 to −0.06; *p* < 0.001) despite similar congestion markers at baseline. The BAN‐ADHF score may be useful clinically to identify patients at risk of diminished response to diuretics.

**What is already known about this subject**

- Diuretic resistance in the context of worsening heart failure is common, and patients with diuretic resistance are at greater risk of adverse events.
- Identifying which patients are unlikely to respond to conventional therapy and develop diuretic resistance is challenging and represents an unmet need in the management of acute HF.
- The recently developed BAN‐ADHF score is a tool that can potentially identify patients with HF at risk of diuretic resistance.

**What this study adds**

- This study provides further multicentre external validation of the BAN‐ADHF score.
- The BAN‐ADHF score is able to identify patients with diminished response to high‐dose loop diuretic treatment.
- Incorporating this score into electronic records could identify patients at risk of diuretic resistance earlier, or be used to select patients who could be managed in an ambulatory care setting.

## 1 INTRODUCTION

The primary goal of treatment in acute heart failure (HF) is to relieve congestion, and this is usually achieved with the use of intravenous loop diuretic. Loop diuretic resistance represents a complex clinical challenge, often leading to prolonged hospitalization, higher readmission rates, worsening renal function and congestion and increased mortality.^1^, ^2^, ^3^ Diuretic resistance is observed in 20%–50% of HF hospitalisations. Treatment includes intensification with adjuvant diuretics, often termed ‘sequential nephron blockade’.^4^, ^5^ Identifying which patients are unlikely to respond to conventional therapy and develop diuretic resistance is challenging and represents an unmet need in the management of acute HF.^3^, ^6^

The recently developed BAN‐ADHF score is a tool that can potentially identify patients with HF at risk of diuretic resistance.^7^ This score was derived and validated in clinical trial datasets.^7^ Higher BAN‐ADHF scores were associated with poorer diuretic responsiveness, greater congestion both at presentation and discharge, longer hospital stays and higher in‐hospital mortality. In a subsequent single‐centre analysis, confirmed higher BAN‐ADHF scores were associated with poorer urine output, weight loss and prognosis.^8^ We sought to provide further external validation of the BAN‐ADHF score in a contemporary multicentre clinical trial of patients with confirmed diuretic resistance.

## 2 METHODS

Dapagliflozin *vs*. metolazone in heart failure resistant to loop diuretics (DAPA‐RESIST) (NCT 04860011) was an open‐label, multicentre randomized trial comparing adjunctive dapagliflozin 10 mg once daily to metolazone 5–10 mg once daily over 96 h in patients hospitalized due to HF who remained congested despite ≥160‐mg intravenous furosemide.^9^ The full protocol, inclusion and exclusion criteria and primary results have been published elsewhere.^9^

A modified BAN‐ADHF score incorporating seven of the original eight variables (blood urea nitrogen, creatinine, natriuretic peptide levels, atrial fibrillation, diastolic blood pressure, hypertension and home diuretic and heart failure hospitalisation) was calculated from baseline data for patients in the DAPA‐RESIST trial; the hypertension component was omitted because hypertension status was not captured. The resulting scores (range 0–23) were dichotomised </≥ the median to define low risk and high risk of diuretic resistance groups used in all subsequent analyses.

### 2.1 Statistical analysis

Baseline characteristics are reported as mean (±SD), median (IQR) or number (%); between‐group differences were examined with Student's *t*, Mann–Whitney *U* or *χ* ^2^ tests, as appropriate. Repeated‐measure trajectories such as the change‐from‐baseline outcomes (weight, 8‐zone B‐line count and modified acetazolamide in acute decompensated heart failure with volume overload [ADVOR] score) were modelled with linear mixed‐effects regression including a random effect for participant and fixed effects for time point, treatment arm (dapagliflozin *vs*. metolazone), BAN‐ADHF risk group (low *vs*. high) and their interaction, left‐ventricular ejection fraction, estimated glomerular filtration rate and recruitment site. This model structure mirrored the analysis approach of the original DAPA‐RESIST trial.

Diuretic response was assessed from baseline to 96 h by measuring mean change in body weight and loop diuretic efficiency (weight change per 40‐mg furosemide). Clinical decongestion over the same interval was evaluated by change in B‐line count on eight‐zone lung ultrasound and a clinical congestion score (modified ADVOR congestion score).^9^, ^10^

Two‐sided *p* < 0.05 denoted statistical significance. All analyses were performed with Stata 18 (StataCorp, College Station, TX, USA).

## 3 RESULTS

### 3.1 Patient and baseline characteristics

All 61 patients from the DAPA‐RESIST trial were included in this analysis. The median BAN‐ADHF score was 8 (IQR 6–12). Baseline demographics and clinical characteristics were similar between the lower and higher BAN‐ADHF score groups (Table 1). There were no statistically significant differences between groups in past medical history. Patients with BAN‐ADHF ≥8 were more likely to have an eGFR <30 mL/min/1.73m^2^ (41% *vs*. 4%; *p* < 0.001). Patients with BAN‐ADHF ≥8 had higher NT‐proBNP concentration (median 6140 pg/mL [IQR 3748–11 758] *vs*. 1792 [1096–3915] pg/mL, *p* < 0.001). Ejection fraction was similar between the two groups. Although there was no statistically significant difference in overall clinical evidence of congestion, more patients with BAN‐ADHF ≥ 8 had ascites and pleural effusions and patients with BAN‐ADHF ≥ 8 were more likely to have experienced prior hospitalization for heart failure. Treatment before admission, and at randomisation, was also similar between groups, other than a significantly higher proportion of patients with BAN‐ADHF score ≥8 were receiving a thiazide diuretic prior to admission, and there was a trend to a higher daily dose of loop diuretic in these patients.

**TABLE 1:** Baseline characteristics according to modified BAN‐ADHF score.

Characteristic	All (*n* = 61)	BAN‐ADHF <8 (*n* = 27)	BAN‐ADHF ≥8 (*n* = 34)	*p*‐value
Age (years)	79 (71–85)	78 (67–83)	81 (74–85)	0.21
Male sex—*n* (%)	28 (46)	10 (37)	18 (53)	0.22
White race—*n* (%)	59 (97)	27 (100)	32 (94)	0.20
BMI (kg/m^2^)	33 (27–37)	32 (27–36)	33 (28–38)	0.05
SBP (mmHg)	116 (106–128)	114 (105–125)	118 (107–129)	0.49
Heart rate (bpm)	72 (66–83)	72 (66–85)	72 (66–82)	0.69
HF history				
Ischaemic aetiology—*n* (%)	20 (33)	6 (22)	14 (41)	0.12
LVEF (%)	45 (35–55)	45 (35–55)	45 (35–55)	0.20
HFrEF—*n* (%)	27 (44)	10 (37)	17 (50)	0.31
Prior HF hospitalization—*n* (%)	35 (57)	13 (48)	22 (65)	0.19
Past medical history—*n* (%)				
Type 2 diabetes	28 (46)	10 (37)	18 (53)	0.22
Myocardial infarction	21 (34)	8 (30)	13 (38)	0.48
Stroke	5 (8)	2 (7)	3 (9)	0.84
AF/flutter	41 (67)	21 (78)	20 (59)	0.12
Peripheral arterial disease	3 (5)	1 (4)	2 (6)	0.70
Physical examination				
Elevated JVP (>4 cm)—*n* (%)	46 (75)	21 (78)	25 (74)	0.70
Pulmonary crepitations—*n* (%)	57 (93)	26 (96)	31 (91)	0.42
Peripheral oedema—*n* (%)	60 (98)	26 (96)	34 (100)	0.26
Ascites—*n* (%)	22 (36)	7 (26)	15 (44)	0.14
Modified ADVOR clinical congestion score	6.0 (5.0–9.0)	6.0 (4.0–7.0)	7.0 (6.0–9.0)	0.14
Pleural effusion^c^—*n* (%)	29 (48)	10 (37)	19 (56)	0.14
Pleural effusion size score	2.0 (2.0–3.0)	2.0 (0.0–3.0)	2.0 (2.0–3.0)	0.42
B‐lines (total number B‐lines)	12.0 (5.5–18.0)	12.0 (4.0–16.0)	14.0 (6.0–18.0)	0.39
Baseline blood tests				
NT‐proBNP (pg/mL)	4052 (1768–6491)	1792 (1096–3915)	6140 (3748–11 758)	<0.001
eGFR (mL/min/1.73m^2^)	41 (32–54)	54 (45–59)	34 (25–41)	<0.001
eGFR <30 mL/min/1.73m^2^—*n* (%)	15 (25)	1 (4)	14 (41)	<0.001
Sodium (mmol/L)	138 (135–140)	138 (137–140)	138 (133–140)	0.44
Potassium (mmol/L)	4.0 (3.8–4.3)	4.0 (3.8–4.2)	4.1 (3.8–4.4)	0.43
Urea (mmol/L)	12.4 (8.3–17.2)	9.3 (7.4–10.9)	16.0 (14.0–20.7)	<0.001
Creatinine (μmol/L)	130 (101–172)	101(88–128)	161 (128–184)	<0.001
HbA1c (mmol/mol)	43.5 (37.0–51.5)	39.0 (37.0–49.0)	46.0 (39.0–54.0)	0.11
Treatment before admission—*n* (%)				
ACEi/ARB/ARNI	19 (31)	9 (33)	10 (29)	0.74
Beta‐blocker	45 (74)	20 (74)	25 (74)	0.96
MRA	22 (36)	9 (33)	13 (38)	0.69
Loop diuretic	54 (89)	23 (85)	31 (91)	0.47
Total Loop diuretic dose (mg)	59 (77)	43 (50)	72 (91)	0.14
Thiazide or thiazide like diuretic	8 (13)	0 (0)	8 (24)	0.01
SGLT2i	2 (3)	0 (0)	2 (6)	0.20
ICD/CRT	3 (5)	0 (0)	3 (9)	0.11
Treatment at randomisation—*n* (%)				
ACEi/ARB/ARNI	14 (23)	9 (33)	5 (15)	0.09
Beta‐blocker	46 (75)	22 (81)	24 (71)	0.33
MRA	22 (36)	11 (41)	11 (32)	0.50
Total daily loop diuretic dose at randomisation (mg)	244 (120)	210 (88)	271 (136)	0.05
Treatment allocated: Dapagliflozin	30 (49)	13 (48)	17 (50)	0.89

*Note*: Values expressed as *n* (%) or median (Quartile 1 and Quartile 3).

^c^ assessed clinically.

Abbreviations: ACEi, angiotensin‐converting enzyme inhibitor; ADVOR, Acetazolamide in Decompensated Heart Failure with Volume Overload; AF, atrial fibrillation; ARB, angiotensin receptor blocker; ARNI, angiotensin receptor blocker neprilysin inhibitor; BMI, body mass index; CKD, chronic kidney disease; CRT, cardiac resynchronisation therapy; eGFR, estimated glomerular filtration rate; HbA1c, haemoglobin A1c; HF, heart failure; ICD, implantable cardioverter defibrillator; JVP, jugular venous pressure; LVEF, left‐ventricular ejection fraction; MRA, mineralocorticoid receptor antagonist; SBP, systolic blood pressure; SGLT2i, Sodium glucose cotransporter‐2 inhibitor.

### 3.2 Diuretic dose, efficiency and serial markers of decongestion

**FIGURE 1:**
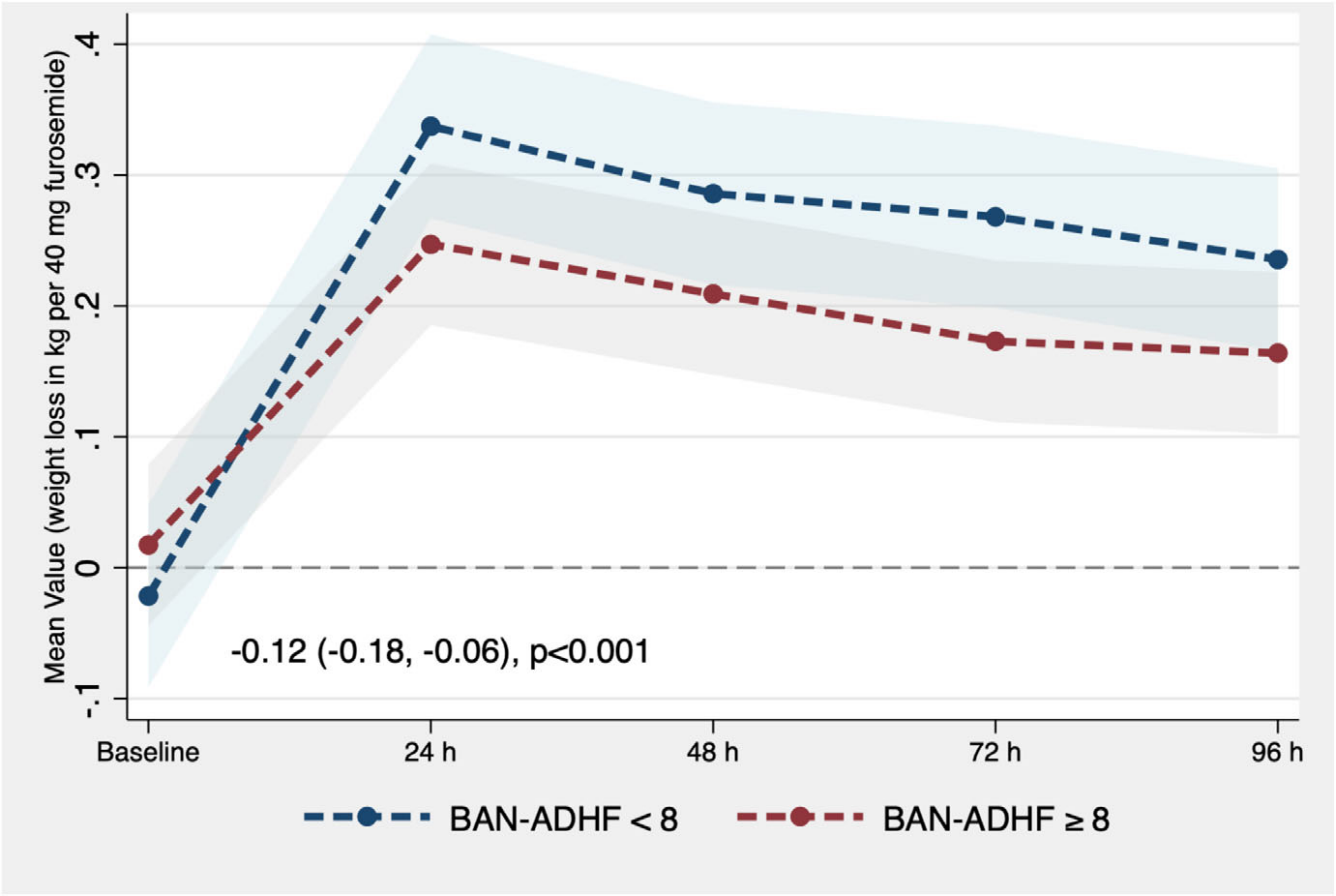
Mean diuretic efficiency, dichotomised by BAN‐ADHF score. For BAN‐ADHF <8 and BAN‐ADHF ≥8 groups, model‐predicted mean change from baseline with 95% confidence intervals is shown for diuretic efficiency at 24‐, 48‐, 72‐ and 96‐h. Diuretic efficiency is defined as weight loss per 40‐mg intravenous furosemide.

**FIGURE 2:**
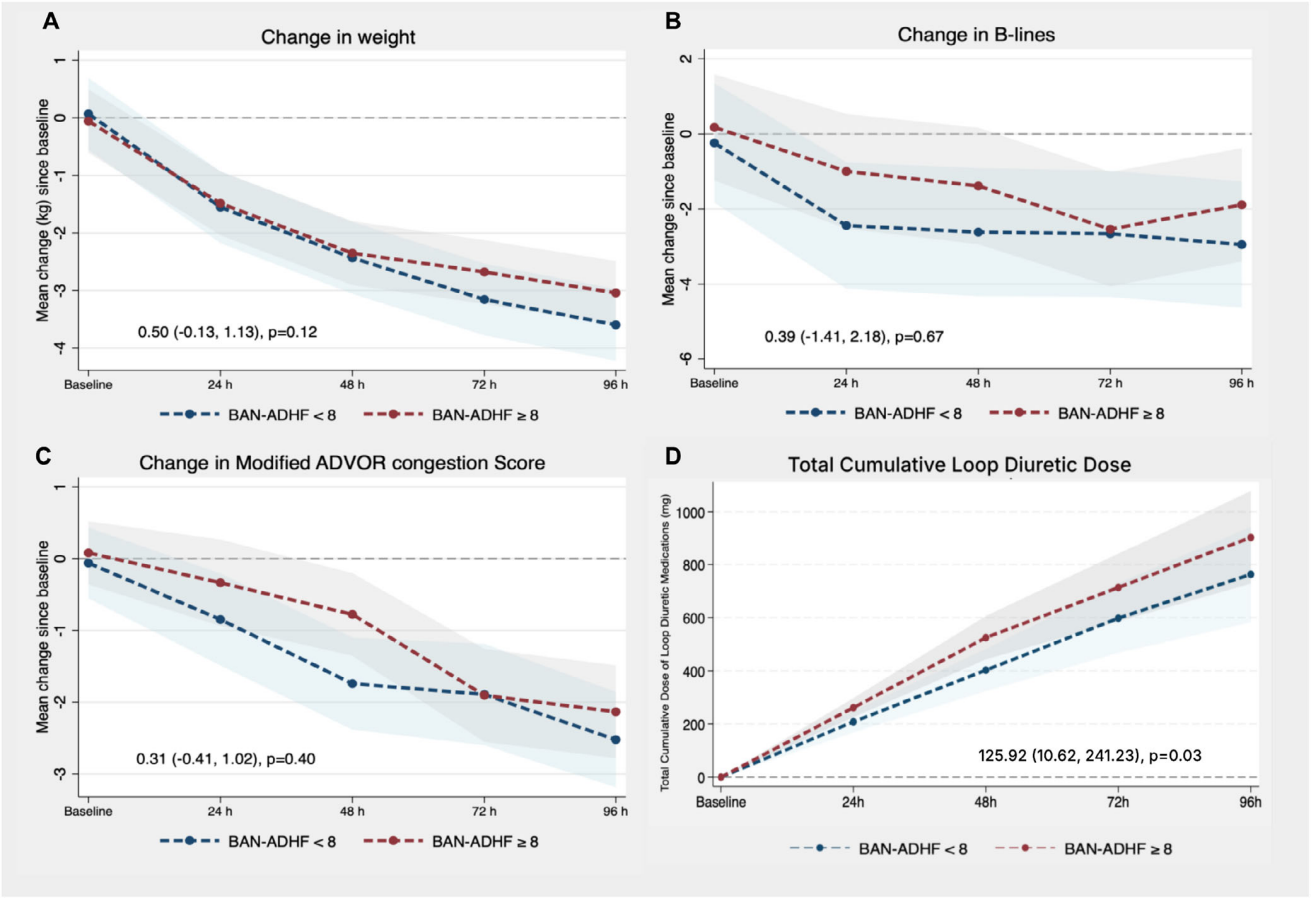
Change in congestion markers and diuretic dose by BAN‐ADHF score. For BAN‐ADHF <8 and BAN‐ADHF ≥8 groups, model‐predicted mean change from baseline with 95% confidence intervals is shown at 24, 48, 72 and 96 h for the following: body weight (kg) (Panel A), B‐lines on lung ultrasound (Panel B) and modified ADVOR congestion score (Panel C). Median cumulative loop diuretic dose with 95% CI (Panel D). The pooled between‐group difference (BAN‐ADHF group ≥8 *vs.* < 8), across the 48–96‐h window is annotated in each panel (Panels A–D).

Loop diuretic efficiency was lower in patients with BAN‐ADHF ≥8 at each time point (Figure 1). The model‐adjusted pooled between‐group difference in loop diuretic efficiency across 48–96 h was −0.12 kg per 40 mg (95% CI –0.18 to −0.06; *p* < 0.001). Total cumulative diuretic dose between 0 and 96 h was numerically greater in the BAN‐ADHF ≥8 group, at 800 (640–960) mg *vs*. 640 (480–960) mg in the <8 BAN‐ADHF group (*p* = 0.13), the model‐adjusted pooled between‐group difference in cumulative loop diuretic dose across 48–96 h was 126 mg (95% CI 11–241; *p* = 0.03) (Figure 2D).

There was no difference in change in body weight between patients with a modified BAN‐ADHF score <8 and ≥8 (Figure 2A). The model‐adjusted between‐group difference in weight change, over the 48–96‐h interval, was 0.50 kg (95% CI –0.13, 1.13; *p* = 0.12). There were no between group differences in BAN‐ADHF categories in congestion as assessed with B‐lines and the modified ADVOR congestion score over the 96‐h treatment period (Figure 2B,C).

## 4 DISCUSSION

Our analysis of the BAN‐ADHF diuretic‐resistance score in DAPA‐RESIST provides multicentre external validation of this score. We have shown that this score is able to identify patients with diminished response to high‐dose loop diuretic treatment. Although investigators in the trial were unaware of this score, they were free to adjust loop diuretic treatment in either the metolazone or dapagliflozin group, as needed, to achieve adequate clinical decongestion. Based on clinical evaluation, they intuitively used a larger total dose of intravenous loop diuretic in patients with a higher BAN‐ADHF score. In so doing, they achieved broadly similar decongestion, although this may have occurred somewhat more slowly, as suggested by Figure 2, and total weight loss was 0.5 kg less in the group with a higher BAN‐ADHF score. These findings suggest that this compensatory clinician dose escalation may have mitigated reduced diuretic efficiency, explaining similar decongestion profiles despite higher BAN‐ADHF scores. While these findings are in no way definitive because of the small sample size and our use of a seven‐variable modified BAN‐ADHF score dichotomised at the cohort's baseline median score, they do raise the possibility that in clinical practice knowledge of a higher likelihood of diuretic resistance (because of a high BAN‐ADHF score) could result in more systematic intensification of diuretic therapy and more careful evaluation of adequacy of decongestion, especially outside the setting of a clinical trial focused on achieving this goal, as was the case in this study. These findings of having higher BAN‐ADHF scores associated with reduced loop diuretic efficiency align with the original publication and another subsequent external validation cohort.^7^, ^8^, ^11^ Our findings are also consistent with the original derivation and subsequent validation cohorts in highlighting the importance of kidney impairment on diuretic resistance, although a higher BAN‐ADHF score was shown to be superior at identifying diuretic resistance than markers of kidney impairment in isolation.^7^ We envisage a potential use case of this score by incorporating it into electronic records, as a flag to clinicians that a patient is at high risk of diuretic resistance. In such a scenario, a patient could be monitored more closely for diminished diuretic response and prompt action taken. A similar system has already been utilized for worsening renal function.^12^ Not only might this score be useful as intended, in highlighting patients with ADHF who might respond poorly to standard intravenous diuretic therapy, but also patients in ambulatory care clinical pathways at high risk of decompensation and potentially avoidable admission to hospital.

## AUTHOR CONTRIBUTIONS

**Dominic M. Alfonso:** Investigation; writing—original draft; formal analysis; data curation; visualization. **Kieran F. Docherty:** Writing—review and editing. **Matthew M. Y. Lee:** Writing—review and editing. **Patrick B. Mark:** Writing—review and editing. **Alex McConnachie:** Data curation; formal analysis. **Joanna Osmanska:** Investigation; data curation; writing—review and editing. **Bethany Stanley:** Data curation; formal analysis. **Atefeh Talebi:** Methodology; formal analysis. **Mark C. Petrie:** Writing—review and editing. **John J. V. McMurray:** Methodology; funding acquisition; supervision; resources; visualization; writing—review and editing. **Ross T. Campbell:** Conceptualization; funding acquisition; writing—review and editing; validation; supervision; resources.

## CONFLICT OF INTEREST STATEMENT

R.T.C. has received consultancy honoraria from Bayer AG and speaking honoraria from AstraZeneca and Circle CVI and has received research grant support from SQ Innovations, Boehringer Ingelheim, Roche Diagnostics and AstraZeneca (paid to his institution). M.C.P. reports research funding from Boehringer Ingelheim, Roche, SQ Innovations, AstraZeneca, Novartis, Novo Nordisk, Medtronic, Boston Scientific, Pharmacosmos and consultancy and trial committee membership for Abbott, Akero, Applied Therapeutics, Amgen, AnaCardio, Biosensors, Boehringer Ingelheim, Corteria, Novartis, AstraZeneca, Novo Nordisk, Abbvie, Bayer, Horizon Therapeutics, Foundry, Takeda, Cardiorentis, Pharmacosmos, Siemens, Eli Lilly, Vifor, New Amsterdam, Moderna, Teikoku, LIB Therapeutics, 3R Lifesciences, Reprieve, FIRE 1, Corvia and Regeneron. M.C.P. is a director of Global Clinical Trial Partners Ltd. K.F.D. reports that his employer, the University of Glasgow, has been remunerated by AstraZeneca and FIRE‐1 for work related to clinical trials; has received speaker honoraria from AstraZeneca, Boehringer Ingelheim, Pharmacosmos, Translational Medicine Academy and Radcliffe Cardiology; has served on an advisory board for Us2.ai and Bayer AG; has served on a clinical endpoint committee for Bayer AG; and has received grant support from Boehringer Ingelheim, Roche Diagnostics, Novartis and AstraZeneca (paid to his institution). P.B.M has received research funding from Boehringer Ingelheim and AstraZeneca, speaker fees from Boehringer Ingelheim, Fresenius, consultancy and trial committee membership from Boehringer Ingelheim, AstraZeneca, Stada UK and Vertex. M.M.Y.L. has received research grants through his institution, the University of Glasgow, from AstraZeneca, Boehringer Ingelheim and Roche Diagnostics; serves on Trial Steering Committees for Amgen and Cytokinetics; and Clinical Endpoint Committees for Bayer and GlaxoSmithKline. J.J.V.M. reports payments to Glasgow University for clinical trials and other research projects from the British Heart Foundation, National Institute for Health—National Heart Lung and Blood Institute (NIH‐NHLBI), Alnylam Pharmaceuticals, AstraZeneca, Bayer, Cardurion, Cytokinetics, Novartis and Roche; personal consultancy fees from Alnylam Pharmaceuticals, AnaCardio, AstraZeneca, Bayer, Cardurion, Cytokinetics, Novartis, River BioMedics, Biohaven Pharmaceuticals, Chugai Pharmaceuticals, Protherics Medicine Developments Ltd. and DalCor Pharmaceuticals; personal lecture fees from Alkem Metabolics, AstraZeneca, Canadian Medical and Surgical Knowledge, Centrix Healthcare, Emcure Pharmaceuticals, Eris Lifesciences, Hikma Pharmaceuticals, Imagica Health, Intas Pharmaceuticals, J.B. Chemicals & Pharmaceuticals, Lupin Pharmaceuticals, Medscape/Heart.Org., ProAdWise Communications, Radcliffe Cardiology, Sun Pharmaceuticals, Translational Medicine Academy, Regeneron, MCI India, Hilton Pharmaceuticals and IMEDIC Pharmaceuticals Micro Labs Ltd. At the Limits Ltd., ARMGO Pharmaceuticals; data safety monitoring boards: WCG Clinical Services. J.J.V.M. is a director of Global Clinical Trial Partners Ltd (which provides clinical trial services such as endpoint committees and educational programmes).

## Data Availability

Trial data will be shared on a reasonable request to the corresponding author.
